# Supplementary material for: BECon: a tool for interpreting DNA methylation findings from blood in the context of brain
Source: Transl Psychiatry. 2017 Aug 1;7(8):e1187–. doi: 10.1038/tp.2017.171 (PMC5611738; doi:10.1038/tp.2017.171)
Supplement: Supplementary Information [file tp2017171x1.pdf]

Supplementary Text

PCA Data Exploration

To decide which, if any, batch effects needed to be adjusted for principal components analysis (PCA) was used to examine the overall variability in the DNAm data. In our PCA the top PC was removed as it is not informative of inter-individual variance in the DNA methylation data[31]. In normal patterns of human methylation data many CpGs typically exist at either a methylated or unmethylated state. For PCA this means the main signal of variability will be shifts in CpG means from one CpG to the next. This top principal component (PC0), is a result of using non-centered beta values in PCA. We therefore adjust our other PCs by the variance accounted for by PC0 in all PCA presented[31].

Of the remaining PCs, we looked at which potential batch variables associated with a given PC and explained some of the methylation variation (Supplementary Figure S2). We based our batch correction on this PCA to variable association.

CpG Concordance

We generated a null distribution of correlations by treating the samples as unpaired. We shuffled the order of the blood samples and ran the correlations calculations again with each brain region five times. The null distribution showed less skewing than the real paired data and allowed us to compare observed potential statistical artifacts of analysis versus true biologically meaningful skewing of the correlation distribution (Supplementary Figure S8).

Explanation of Variability and Correlation Thresholds

We found that the most variable and highly positively correlated CpGs were those with a known SNP at the cytosine or guanine of a CpG (polymorphic CpGs) and those on the sex chromosomes. These CpGs represented highly informative sites between blood and brain, as gender and genotype differences would be generally consistent between tissues. We therefore used these sites to inform our threshold of variability and correlation of other CpGs. The threshold we used for variability was informed by the emergence of a peak of highly correlated highly variable CpGs which emerged as more variable CpGs were selected (Supplementary Figure S12). This peak represents many of the polymorphic and sex chromosome CpGs. To define where this highly correlated peak separates from the main peak, we used a beta mixture model to fit two beta mixtures to the bimodal correlation distribution of all probes with more than 0.25 reference range variability (Figure 3C). The correlation threshold was set for each Brodmann area individually. The thresholds were set at the mean of the positively correlated peak minus 2 standard deviations. The inverse of theses correlation thresholds were also used to define a set of negatively correlated CpGs. We have analysed the negatively and positively correlated CpGs identically but separately.

The threshold for variability was also informed from the SNP-CpGs and sex chromosome CpGs. The mean reference range of these sites is 0.111 so a threshold of 0.1 was used. Conveniently, 0.1 is a common threshold for delta

beta in differential methylation studies so is a generally accepted threshold for biologically meaningful difference in methylation.

CpG to Gene Annotation

We used a CpG to gene association definition described previously[46]. Refseq genes were downloaded from UCSC, including all isoforms of a gene. The gene list included 24 047 genes and a total of 33 431 unique transcription units. If the CpG is 1500bp upstream of a gene's TSS or 300 downstream it is considered to be in the genes promoter. If the CpG is 300bp downstream of the genes TSS to 300 bp upstream of the genes end then it is considered in a genes body (intragenic). If the CpG is 300 bp +/- the gene end it is in the 3' region. Importantly, there is a 4th category which closest TSS does not take into account, if a CpG is in none of the above categories it is considered intergenic and not directly associated with a gene. The 485 512 CpGs on the 450K array associated with 23,018 genes (43.8% intragenic, 34.2% promoter, 2.5% 3 region, 19.5% intergenic).

Functional Exploration of Informative CpGs

GO enrichment

Using the list of 40, 029 informative CpGs (positively and negatively correlated) we generated a list of genes which are expected to be informative between blood and brain. The list was generated by selecting genes which had greater than ten informative CpGs associated. This list of 239 genes was used for functional enrichment of Gene Ontology (GO) terms. For enrichment analysis we used a background list of the GO annotations of the 8 537 genes associated with at least ten 450K CpGs. Enrichment of GO terms in the list of informative genes was tested using overrepresentation analysis in ErmineJ[61]. Statistical significance is reported as false discovery rates computed using the Benjamini-Hochberg method in ErmineJ. Also calculated are the multifunctionality p-values of the ontology gene sets[50].

Gene Expression Analysis

To examine if our informative genes were more highly expressed generally in blood or brain we collected unrelated samples of gene expression data from human blood and brain from GEO. For the brain expression we used GSE17612, which is a dataset of 51 samples from Brodmann Brain area 10, for the blood expression there were many more samples available so we used a data set of 70 samples from 2 studies, GSE37171 and GSE61635. Of the 239 informative genes 189 were represented on the gene expression array used (GPL570). The expression level of the 189 informative genes was compared to the expression level of 189 random genes in 100 permutations using a Wilcoxon Rank Sum test.

BECon Development

Using R shiny (version 0.13.2) we built an web application to view our data (BECon). The application allows searching from 23 018 Refseq gene names and 423 384 CpG IDs (those which passed quality control). BECon displays line plots of the methylation beta values in each sample across individuals,

to visualize trends in the actual methylation data at the CpGs selected. A text line gives information on how many CpGs selected passed quality control in our dataset. Displayed below the line plots is a heat map with summary statistics for each CpG selected. The heat map first shows the basic genomic information: chromosome, genomic coordinate, genes associated with the CpG and gene region. The green columns show the reference range variability metric for each CpG in each tissue, boxes in darker green pass a 0.05 reference range threshold. In grey and yellow the correlation of DNAm between blood and each brain region is shown, the color of these boxes is related to the percentile rank of the correlation score of a CpG within each tissue. Finally the columns labelled cell composition display a metric of the mean change in DNAm beta value before and after cell composition adjustment, as a measure of the effect of cell composition on each CpG in each tissue. The colors represent the percentile rank of the change with cell composition with darker colors representing greater effects of cell composition.

Supplementary Figures

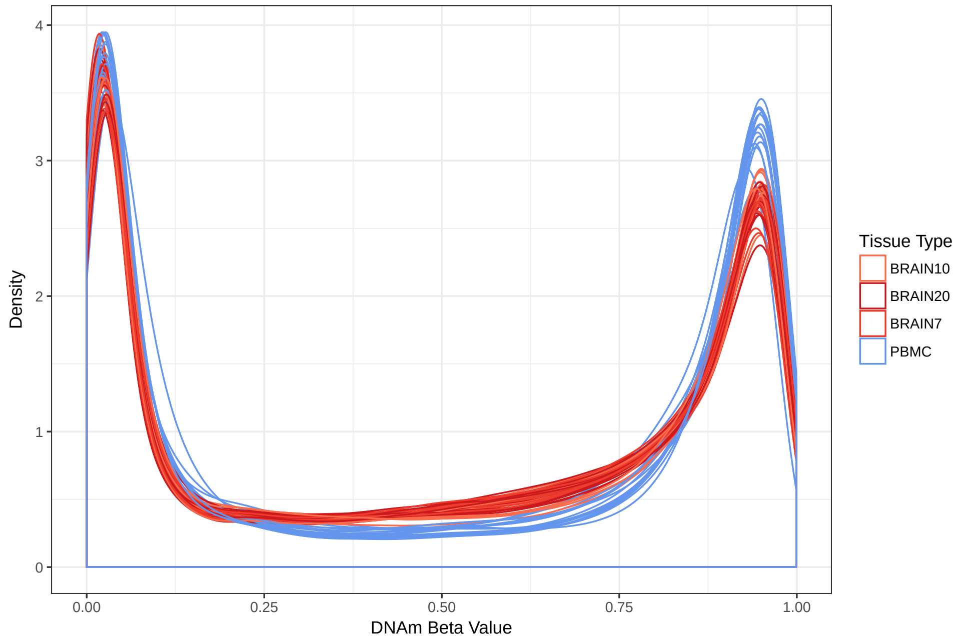

Figure S1: Distribution of DNAm beta values for each sample. Samples are coloured by tissue.

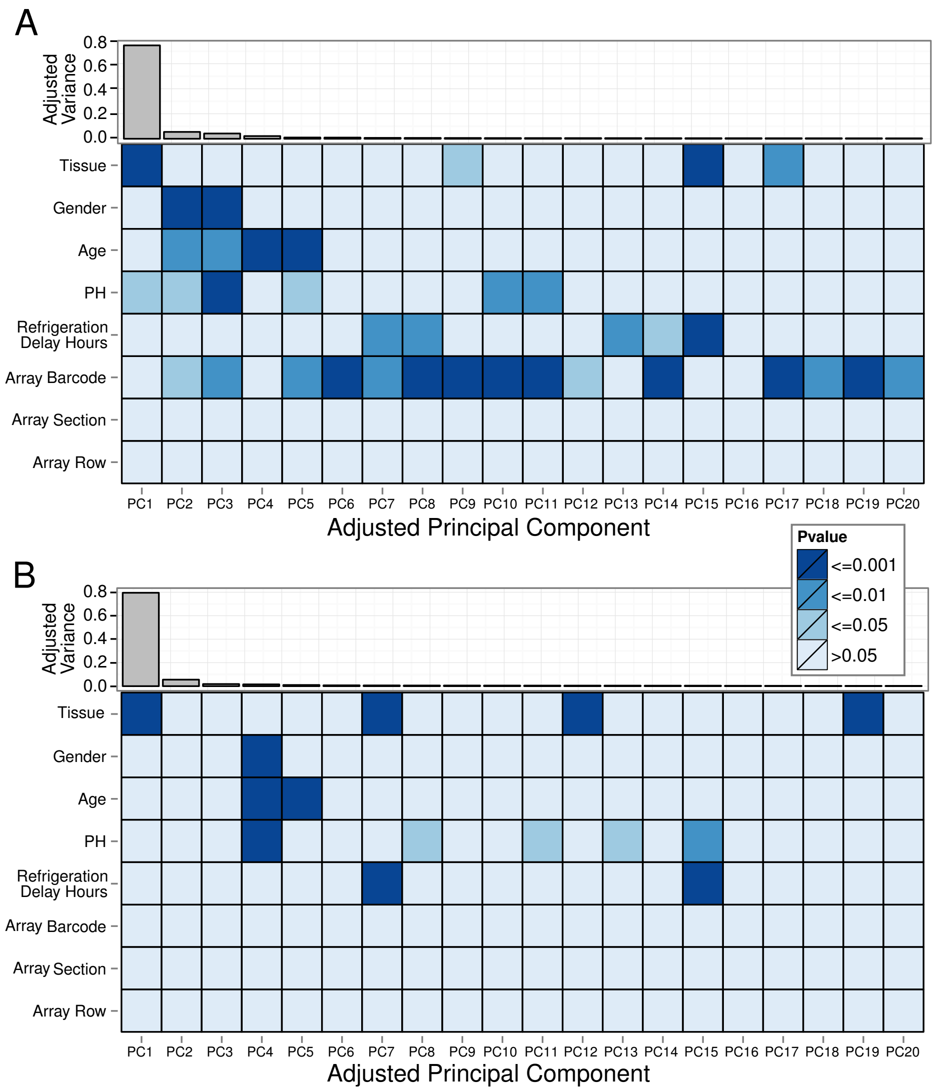

Figure S2: PCA to examine which meta data variables should be adjusted for using ComBat. Scree plots show the amount of methylation variance (adjusted for PC0) accounted for by each PC. Heat maps show the associations (correlation p value for continuous variables; ANONA p value for categorical variables) between a meta data variable and an individual PC. A) before ComBat B) After ComBat, PCA was rerun and the scree plot and heat map show the association with the new PCs.

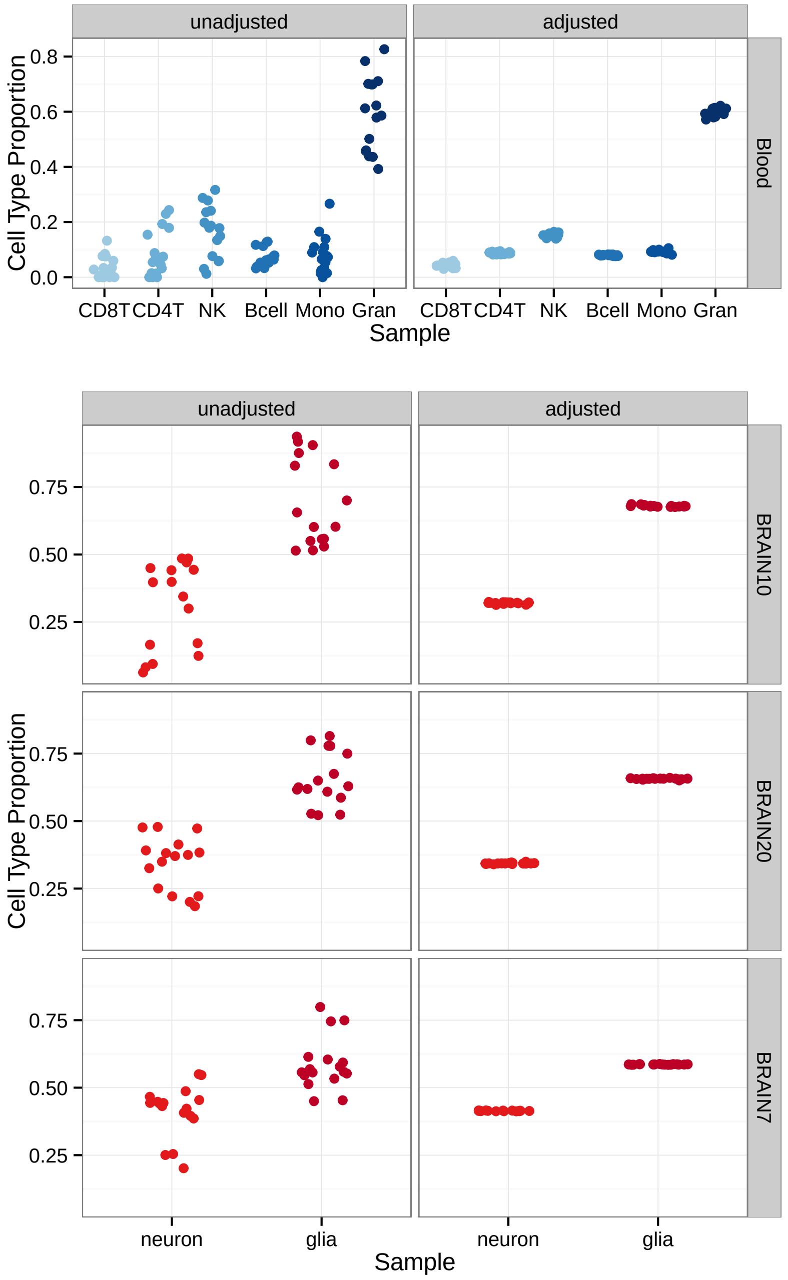

Figure S3: Proportions of cell types in blood and brain varied between samples before normalization. A) The plot on the left shows the estimated blood cell proportions before blood cell type normalization and on the right the estimate proportions after normalization. B) Similar to A but for brain cell type correction in the brain samples, each brain region was normalized separately.

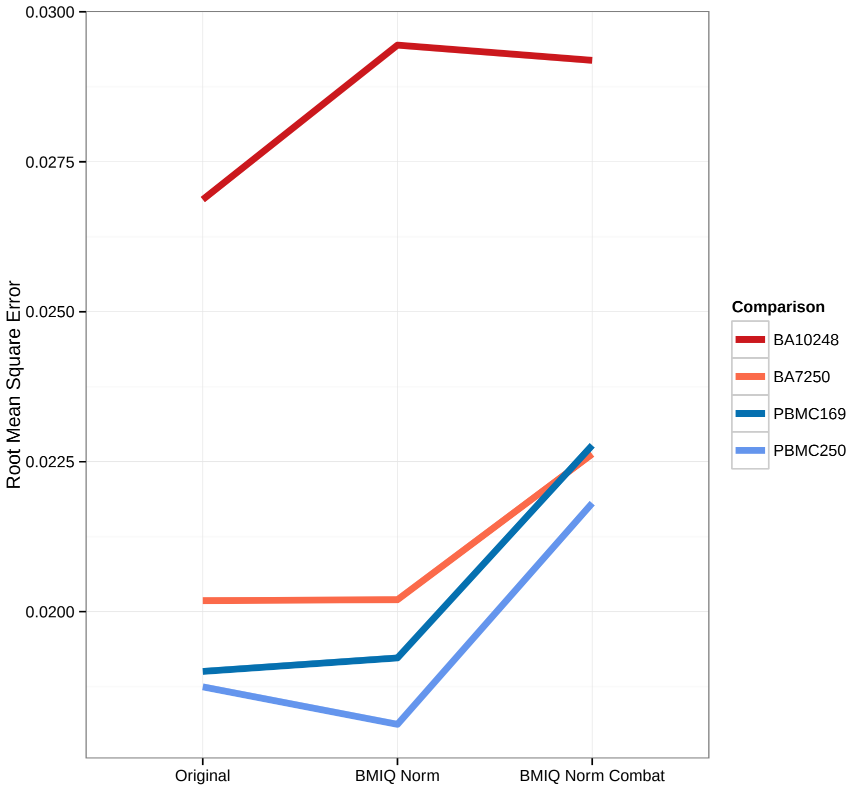

Figure S4: Replicated pairs show low RMSE at each processing step. Root mean square error values are shown on the y and the stage of preprocessing is shown on the x axis, raw DNAm values, BMIQ normalized and normalized and batch corrected DNAm values.

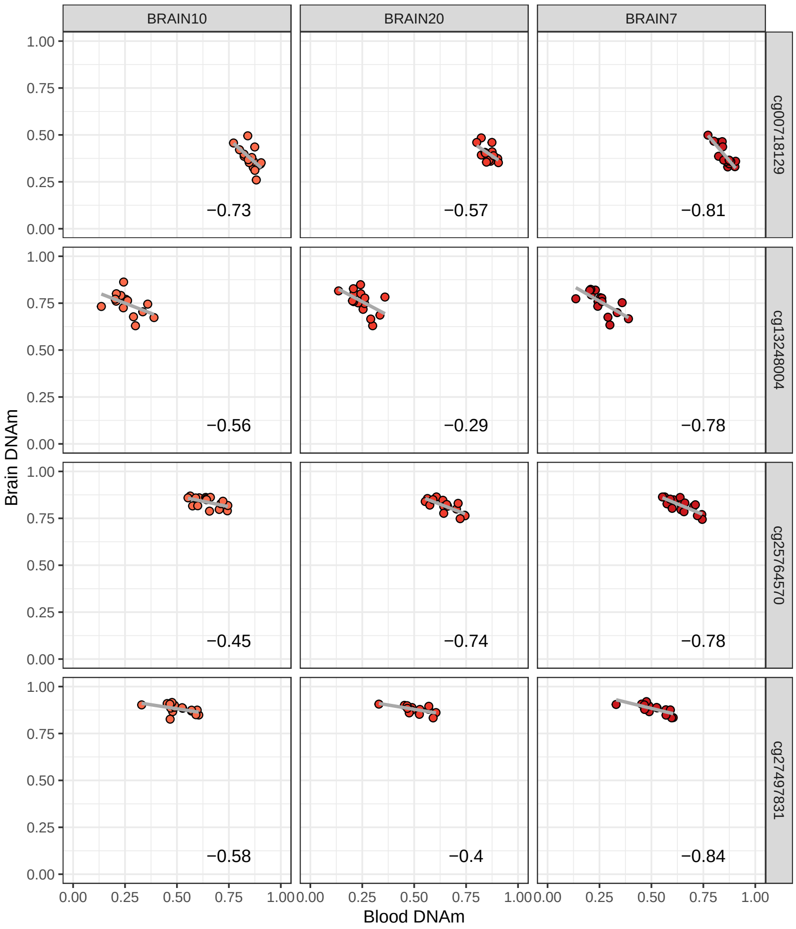

Figure S5: Representative CpGs of negative correlation between blood and brain. For each CpG a plot is shown for each brain region with blood DNAm on the x axis. Spearman correlations are shown for the correlation between blood DNAm and each Brodmann area DNAm

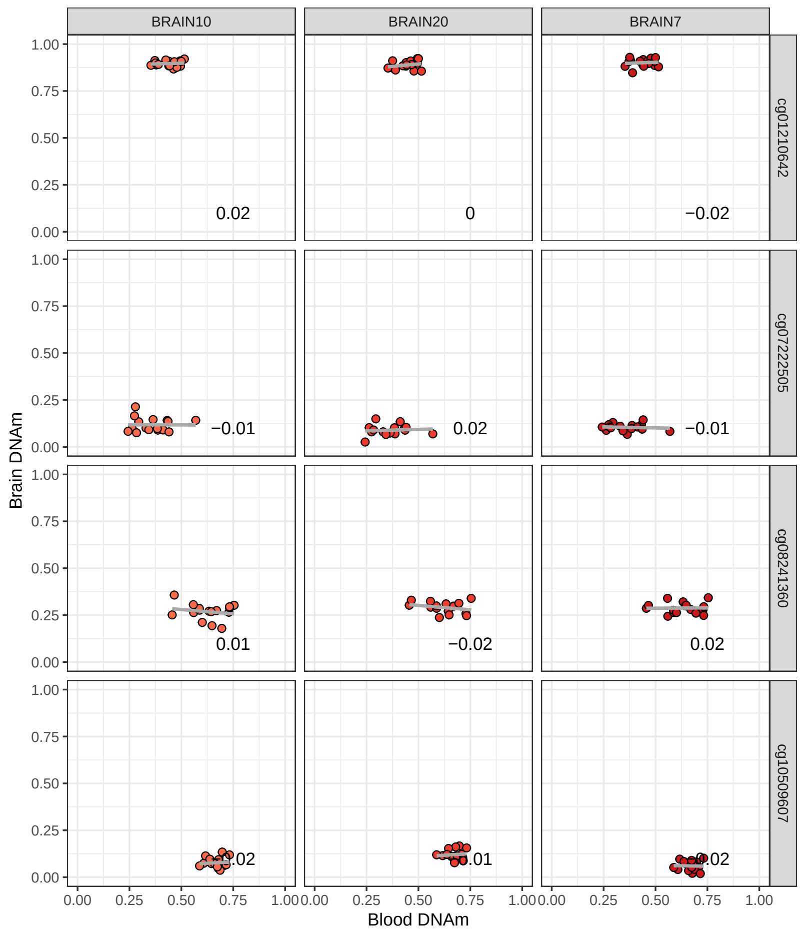

Figure S6: Representative CpGs of no correlation between blood and brain. For each CpG a plot is shown for each brain region with blood DNAm on the x axis. Spearman correlations are shown for the correlation between blood DNAm and each Brodmann area DNAm

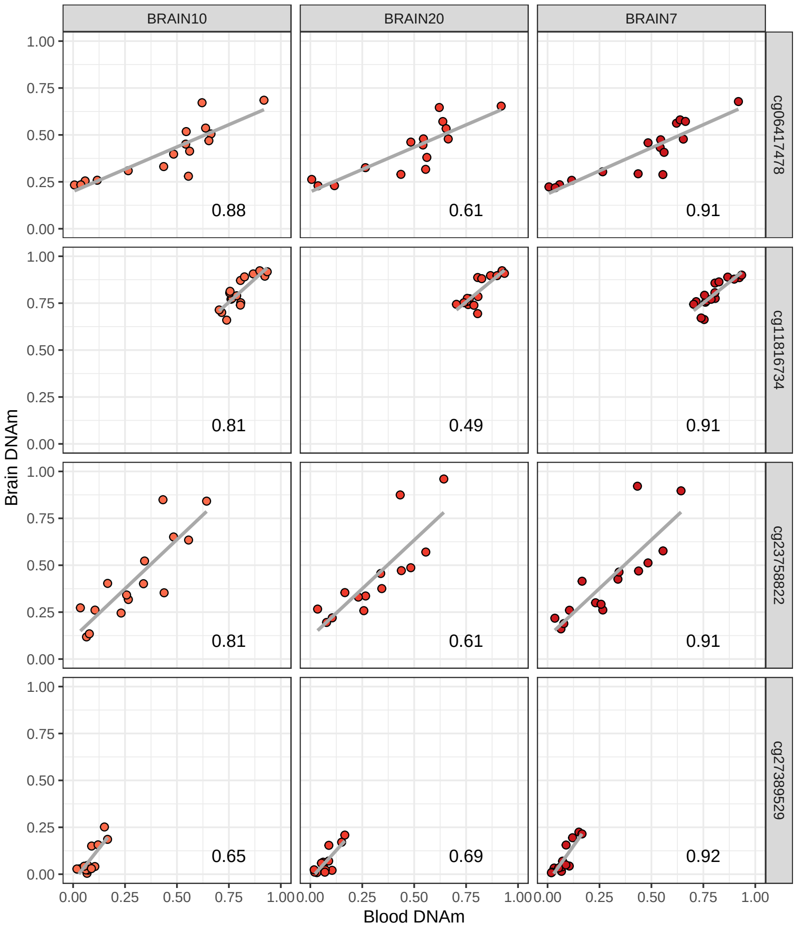

Figure S7: Representative CpGs of positive correlation between blood and brain. For each CpG a plot is shown for each brain region with blood DNAm on the x axis. Spearman correlations are shown for the correlation between blood DNAm and each Brodmann area DNAm

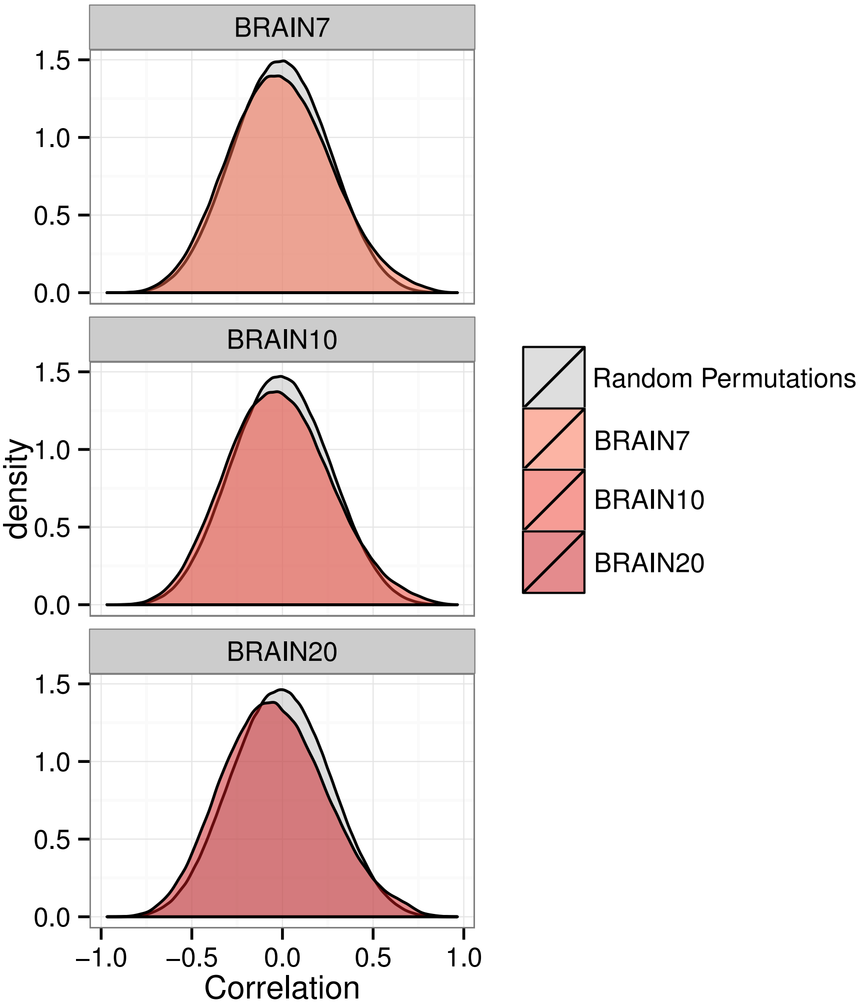

Figure S8: Correlation distributions of paired and unpaired permutations. The filled grey density curves show the random permutations of unpaired samples, while the filled red curves show the correlation distribution for the paired samples.

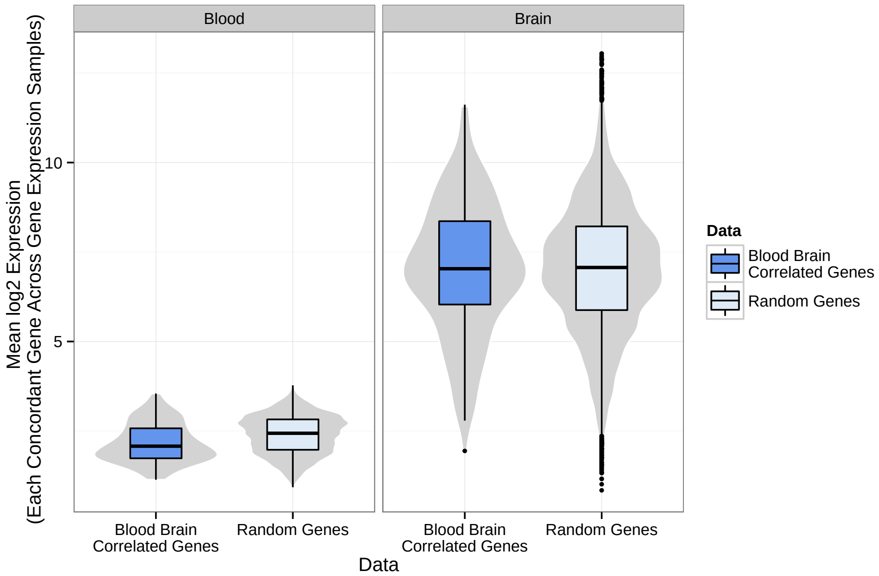

Figure S9: Genes associated with blood and brain informative CpGs show different levels of expression in independent blood and brain samples. Mean gene expression across samples of 189 informative genes (dark blue) or a random list of 189 genes (light blue) in shown in the boxplots.

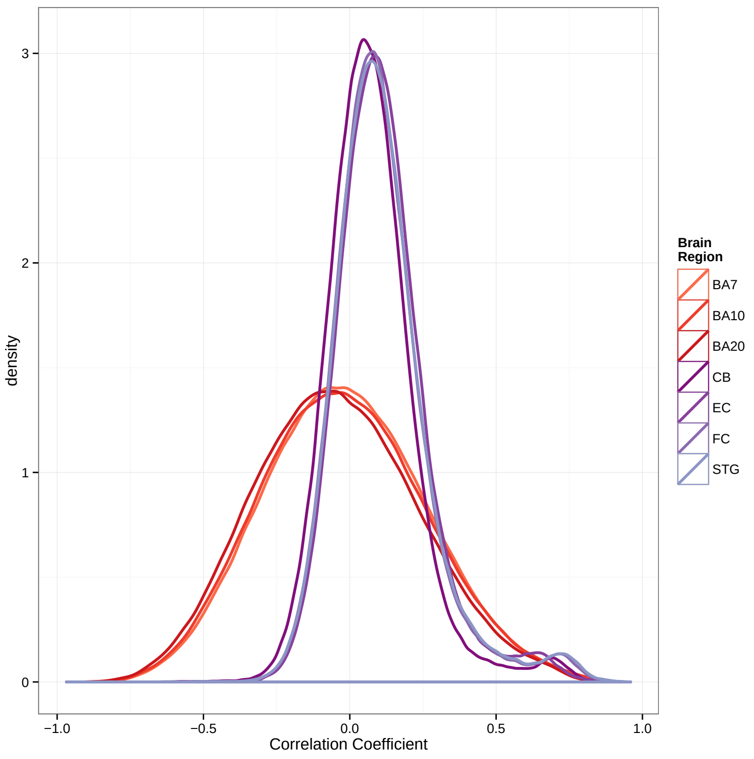

Figure S10: Sample size effects the correlation distribution in studies of blood and brain. The correlation distributions for our three brain regions are shown in red and orange and the correlation distributions from the four brain regions in[33] are shown in purple.

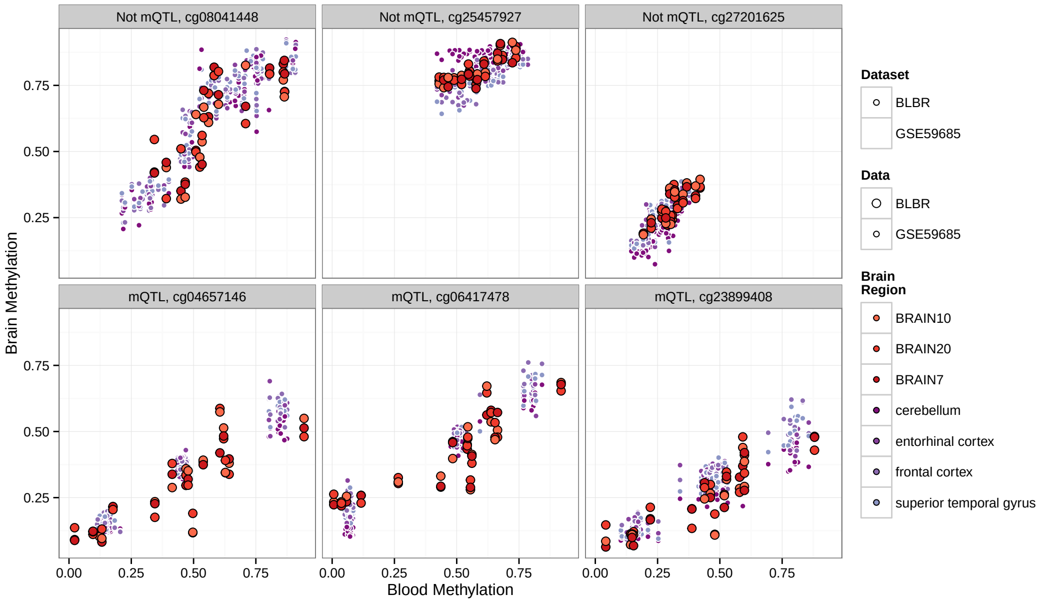

Figure S11: Representative CpGs selected to show examples of CpGs that were characterized as informative in both our study (red and orange points) and the[33] work (purple points) but were not seen as an mQTL in either study (top three examples). Representative CpGs were also selected that show cases where in the larger population the CpG can be identified as a potential mQTL but the pattern is not clear in our smaller sample size (bottom three examples).

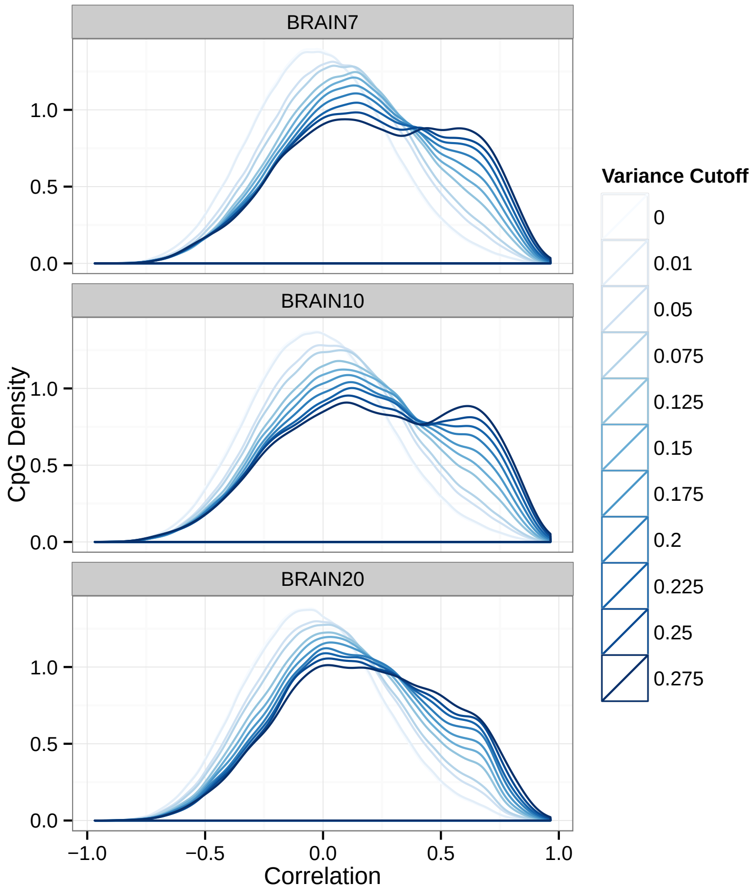

Figure S12: A highly positively correlated peak is seen when SNP-CpG and sex chromosome CpGs are included in the correlation distributions. Correlation distributions are shown for each brain region. Line colours darken as the CpGs underlying the distribution become more strictly thresholded on reference range.

Supplementary Tables

Table S1: The number of informative CpGs at various thresholds of correlation and reference range. The columns are possible reference range thresholds, and rows are the possible correlation thresholds.

		**Reference Range Threshold**		
	**Brodmann Area 7**	**0.05**	**0.1**	**0.2**
	**0.3**	29 037	17 789	4 447
**Positive Correlation**	**0.4**	15 749	10 102	2 799
	**0.5**	7 683	5 156	1 610
	**0.8**	105	121	69
	**-0.3**	18 995	8 090	1 463
**Negative Correlation**	**-0.4**	9 350	3 847	721
	**-0.5**	3 908	1 566	309
	**-0.8**	26	7	0
		**Reference Range Threshold**		
	**Brodmann Area 10**	**0.05**	**0.1**	**0.2**
	**0.3**	27 924	17 067	4 451
**Positive Correlation**	**0.4**	15 487	9 858	2 879
	**0.5**	7 751	5 172	1 706
	**0.8**	173	137	74
	**-0.3**	20 800	9 549	1 704
**Negative Correlation**	**-0.4**	10 328	4 743	872
	**-0.5**	4 485	2 081	382
	**-0.8**	25	10	3
		**Reference Range Threshold**		
	**Brodmann Area 20**	**0.05**	**0.1**	**0.2**
	**0.3**	23 742	14 138	3 717
**Positive Correlation**	**0.4**	12 707	7 803	2 213
	**0.5**	6 106	3 898	1 193
	**0.8**	109	81	47
	**-0.3**	25 577	11 807	1 893
**Negative Correlation**	**-0.4**	13 094	5 945	933
	**-0.5**	5 809	2 535	394
	**-0.8**	52	17	6

Table S2: Top over-represented GO groups in the genes informative between blood and brain. Columns are: name of the GO gene set, GO ID, Multifunctionality p-value, nominal over representation p value and Benjamini-Hochberg corrected over representation p value (which the data is sorted by)

**Name**	**ID**	**Multifunctionality p-value**	**p-value**	**Corrected p-value**
homophilic cell adhesion via plasma membrane adhesion molecules	GO:0007156	0.000791	5.82E-17	1.73E-13
cell-cell adhesion via plasma membrane adhesion molecules	GO:0098742	0.00113	1.52E-15	2.26E-12
genetic imprinting	GO:0071514	0.367	5.01E-05	0.0497456
single-organism behavior	GO:0044708	0.922	4.76E-04	0.35391988
cellular response to glucagon stimulus	GO:0071377	0.422	8.42E-04	0.50147476
response to glucagon	GO:0033762	0.56	1.53E-03	0.75768702

Table S3: CpGs identified as differentially methylated in blood based studies of neurobiological disease.

Gene	Differentially Methylated CpGs	Outcome Phenotype	Tissue	Author	Pubmed ID	Year	Method
DRD4	cg03909863	Schizophrenia	peripheral blood	Cheng et al.	24586542	2014	Pyrosequencing
BDNF	cg16257091	Major Depression	peripheral blood	Fuchikami et al.	21912609	2011	SEQUENOM
COMT	cg22546130, cg23601416	schizophrenia	leukocytes	Melas et al	22426120	2012	Pyrosequencing
OXTR	cg03987506, cg09353063	Acute psychosocial stress	whole-blood cells	Unternaehrer et al.	22892716	2012	SEQUENOM

Table S4: Correlation of CpGs identified as differentially methylated in blood based studies of neurobiological disease between blood and brain. The table is in the same format as that provided in the R Shiny application for viewing CpGs of interest. Columns are: CpG ID on the 450K; Genomic Coordinate; Chromosome; Genes Associated with the CpG; Feature of the gene in which the CpG is present (multiple features for some genes because of isoform size differences); Correlation Between Blood and Brodmann Brain Area 7; 10; and 20; Mean correlation in all three brain regions; Percentile of correlation value compared to all CpGs, if positive; Percentile of correlation value compared to all CpGs, if negative; Reference range variability in Blood; Brodmann brain area 7; 10; 20

CpG ID	Genomic Coordinate (hg19)	Chr (hg19)	Associated Genes	CpG in Feature of Gene, respectively	Cor Blood-BA7	Cor Blood-BA10	Cor Blood-BA20	Mean Cor All Brain	SD Mean Cor All Brain	Percentile of Mean Cor All Brain (positive)	Percentile of Mean Cor All Brain (negative)	Var in Blood	Var in All Brain	Var in BA7	Var in BA10	Var in BA20
cg03909863	638404	11	DRD4	intragenic	0.41	0.6	0.45	0.49	0.1	90%	-	0.13	0.14	0.11	0.11	0.15
cg03987506	8810549	3	OXTR	intragenic	-0.12	-0.13	0.39	0.05	0.3	<50%	-	0.03	0.05	0.04	0.05	0.04
cg09353063	8811092	3	OXTR	promoter	-0.17	0.15	-0.42	-0.15	0.29	-	50-75%	0.03	0.02	0.02	0.02	0.02
cg16257091	27743580	11	BDNF	promoter	0.11	-0.08	0.16	0.06	0.12	<50%	-	0.04	0.04	0.04	0.04	0.04
cg22546130	19950026	22	COMT, COMT, MIR4761	intragenic, promoter, promoter	0.54	0.26	-0.01	0.26	0.28	75-90%	-	0.16	0.16	0.09	0.2	0.1
cg23601416	19950040	22	COMT, COMT, MIR4761	intragenic, promoter, promoter	0.45	0.09	-0.09	0.15	0.27	50-75%	-	0.13	0.1	0.08	0.13	0.08
